# Luminescent zero-dimensional organic metal halide hybrids with near-unity quantum efficiency†
†Electronic supplementary information (ESI) available. CCDC 1497737, 1580794 and 1538293. For ESI and crystallographic data in CIF or other electronic format see DOI: 10.1039/c7sc04539e


**DOI:** 10.1039/c7sc04539e

**Published:** 2017-11-21

**Authors:** Chenkun Zhou, Haoran Lin, Yu Tian, Zhao Yuan, Ronald Clark, Banghao Chen, Lambertus J. van de Burgt, Jamie C. Wang, Yan Zhou, Kenneth Hanson, Quinton J. Meisner, Jennifer Neu, Tiglet Besara, Theo Siegrist, Eric Lambers, Peter Djurovich, Biwu Ma

**Affiliations:** a Department of Chemical and Biomedical Engineering , FAMU-FSU College of Engineering , Tallahassee , FL 32310 , USA . Email: bma@fsu.edu; b Materials Science and Engineering Program , Florida State University , Tallahassee , FL 32306 , USA; c Department of Chemistry and Biochemistry , Florida State University , Tallahassee , FL 32306 , USA; d National High Magnetic Field Laboratory , Florida State University , Tallahassee , FL 32310 , USA; e Research Service Centers , University of Florida , Gainesville , Florida 32661 , USA; f Department of Chemistry , University of Southern California , Los Angeles , California 90089 , USA

## Abstract

Single crystalline zero-dimensional (0D) organic–inorganic hybrid materials with perfect host–guest structures have been developed as a new generation of highly efficient light emitters. Here we report a series of lead-free organic metal halide hybrids with a 0D structure, (C_4_N_2_H_14_X)_4_SnX_6_ (X = Br, I) and (C_9_NH_20_)_2_SbX_5_ (X = Cl), in which the individual metal halide octahedra (SnX_6_^4–^) and quadrangular pyramids (SbX_5_^2–^) are completely isolated from each other and surrounded by the organic ligands C_4_N_2_H_14_X^+^ and C_9_NH_20_^+^, respectively. The isolation of the photoactive metal halide species by the wide band gap organic ligands leads to no interaction or electronic band formation between the metal halide species, allowing the bulk materials to exhibit the intrinsic properties of the individual metal halide species. These 0D organic metal halide hybrids can also be considered as perfect host–guest systems, with the metal halide species periodically doped in the wide band gap matrix. Highly luminescent, strongly Stokes shifted broadband emissions with photoluminescence quantum efficiencies (PLQEs) of close to unity were realized, as a result of excited state structural reorganization of the individual metal halide species. Our discovery of highly luminescent single crystalline 0D organic–inorganic hybrid materials as perfect host–guest systems opens up a new paradigm in functional materials design.

## Abstract 2

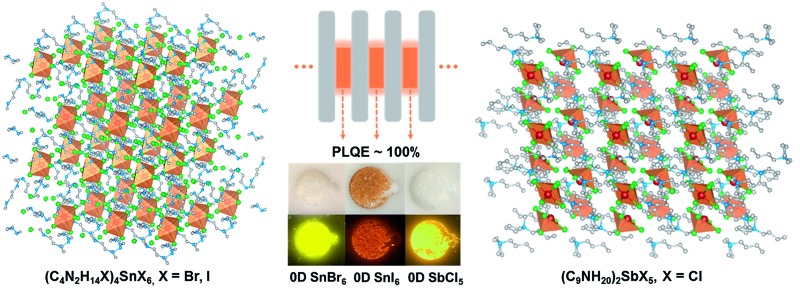
Single crystalline zero-dimensional organic metal halide hybrids have been developed.

## Introduction

Light emitting materials are one of the essential components of life today with applications in a wide range of areas, from energy to information, environmental and healthcare technologies. Various types of light emitting materials have been developed over time, including organic and polymeric emitters, transition metal complexes, rare-earth doped phosphors, nanocrystals and organic–inorganic hybrid perovskites.^1^–^6^ One key design used to realize highly efficient light emitting materials and devices is the host–guest concept,^7^ in which the light emitting species are doped in an inert host matrix. The benefits of the host–guest design are multifold, *i.e.* suspending aggregation induced self-absorption and self-quenching, as well as allowing for facile fine-tuning of the emission color. However, to realize highly efficient host–guest systems with guests uniformly distributed in a host matrix is not trivial, as it requires careful selection of the host and guest materials, as well as precise control of the material processing for an optimized doping concentration. One promising approach to obtaining perfect light emitting host–guest systems is the formation of single crystalline bulk assemblies of 0D structured materials, in which the light emitting species are periodically embedded in a host matrix and completely isolated from each other, without electronic band formation.

A crystalline solid is a material whose constituents, such as atoms, molecules or ions, are arranged in an ordered structure, forming a periodic lattice that extends in all directions. The interactions between the lattice points could lead to the formation of electronic band structures.^8^ As a result, the properties of inorganic crystals show a strong dependence on their size, especially in the nanoscale, the so called quantum size effect.^9^ The molecular interactions in organic crystals cause their properties to be distinct from those of individual molecules.^10^ Single crystalline materials that exhibit bulk properties consistent with their individual building blocks, or bulk assemblies of 0D materials without electronic band formation or quantum size effects, are rare to the best of our knowledge. In the present work, we have synthesized and characterized single crystalline bulk assemblies of 0D hybrid materials as perfect host–guest systems, (C_4_N_2_H_14_X)_4_SnX_6_ (X = Br or I) and (C_9_NH_20_)_2_SbX_5_ (X = Cl), which exhibit Gaussian-shaped and strongly Stokes shifted broadband emissions, with PLQEs of close to unity. Note that our 0D hybrid materials are bulk crystals with 0D structures on a molecular level, not morphological nanoscale 0D materials, such as quantum dots.

## Results and discussion

**Fig. 1:**
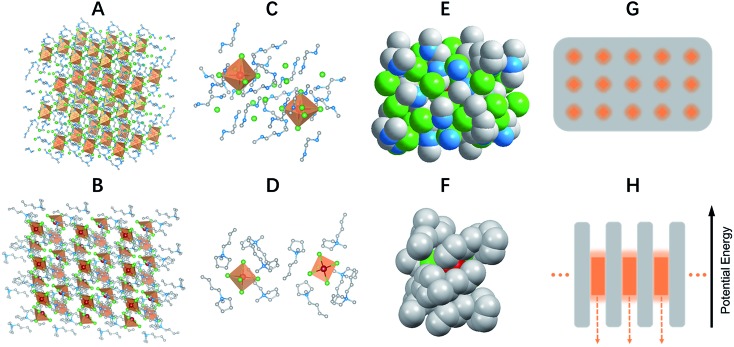
Single crystal structure and energy diagram of 0D organic metal halide hybrids. Views of the structures of (C_4_N_2_H_14_Br)_4_SnBr_6_ (A) and (C_9_NH_20_)_2_SbCl_5_ (B) (red spheres: metal centers; green spheres: halide atoms; blue spheres: nitrogen atoms; gray spheres: carbon atoms; orange polyhedrons: SnBr_6_^4–^ octahedra and SbCl_5_^2–^ quadrangular pyramids; hydrogen atoms were hidden for clarity). Views of two SnBr_6_^4–^ octahedra completely isolated from each other and surrounded by C_4_N_2_H_14_Br^+^ ligands (C) and two isolated SbCl_5_^2–^ quadrangular pyramids surrounded by C_9_NH_20_^+^ ligands (D). Space filling models with an individual SnBr_6_^4–^ completely covered by C_4_N_2_H_14_Br^+^ ligands (E) and an individual SbCl_5_^2–^ covered by C_9_NH_20_^+^ ligands (F). Schematic drawing of a perfect host–guest system with the light emitting species periodically embedded in an inert matrix (G), and its potential energy diagram (H).

Single crystalline (C_4_N_2_H_14_X)_4_SnX_6_ (X = Br or I) was prepared by slowly diffusing dichloromethane into a dimethylformamide or γ-butyrolactone precursor solution of tin halide (SnX_2_, X = Br or I) and *N*,*N*′-dimethylethylene-1,2-diammonium halide (CH_3_NH_2_^+^CH_2_CH_2_NH_2_^+^CH_3_·2X^–^) at room temperature in a N_2_ filled glove box. (C_9_NH_20_)_2_SbCl_5_ was prepared by slowly diffusing acetone into a dimethylformamide precursor solution of antimony trichloride (SbCl_3_) and 1-butyl-1-methylpyrrolidinium chloride (C_9_NH_20_^+^·Cl^–^). The crystal structures of these 0D organic metal halide hybrids were determined using single crystal X-ray diffraction (SCXRD) (Table S1†), and show a 0D structure with the individual metal halide ions, SnX_6_^4–^ and SbX_5_^2–^, completely isolated from each other and surrounded by the large organic ligands, C_4_N_2_H_14_X^+^ and C_9_NH_20_^+^, respectively (Fig. 1A and B). It is worth pointing out that (C_4_N_2_H_14_X)_4_SnX_6_ (X = Br or I) could indeed be considered as a true 0D organometal halide perovskite.^11^,^12^ The complete isolation of the photoactive metal halide species by the wide band gap organic ligands, with the distance between two metal centers being >1 nm as shown in Fig. 1C and D, leads to no interaction between the photoactive metal halide species or electronic band formation. Fig. 1E and F clearly show the full coverage of organic ligands on the individual metal halide species in a space-filling model, suggesting a perfect 0D core–shell structure. Therefore, the potential energy diagram for these bulk assemblies of 0D materials, or perfect host–guest systems (Fig. 1G), can be described as in Fig. 1H, and enables the bulk materials to exhibit the intrinsic properties of the individual metal halide species. The powder X-ray diffraction (PXRD) patterns of the ball-milled crystal powders display exactly the same features as the simulated patterns from SCXRD (Fig. S1†), suggesting a uniform crystal structure of the as-synthesized 0D organic metal halide hybrids. Elemental analysis also confirmed the purity and uniformity of these materials. To further verify the structure, composition, and that only Sn(ii) was present in the Sn based materials, solid state ^119^Sn nuclear magnetic resonance (NMR) spectroscopy (Fig. S2†) and X-ray photoelectron spectroscopy (XPS) (Fig. S3†) measurements were performed.

**Fig. 2:**
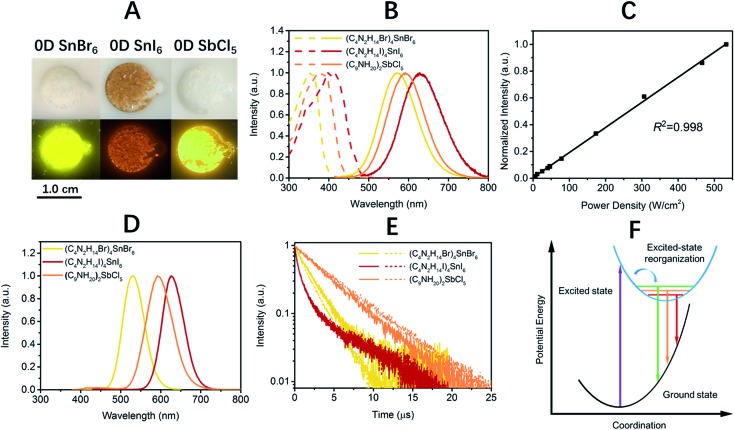
Photophysical properties of 0D organic metal halide hybrids at room temperature and 77 K. Images of 0D organic metal halide hybrids under ambient light and UV lamp irradiation (365 nm) (A). Excitation (dashed lines) and emission (solid lines) spectra of 0D organic metal halide hybrids at room temperature (B). Emission intensity *versus* excitation power for (C_4_N_2_H_14_Br)_4_SnBr_6_ at room temperature (C). Emission spectra of 0D organic metal halide hybrids at 77 K (D). The emission decay of 0D organic metal halide hybrids at room temperature (solid lines) and 77 K (dashed lines) (E). The mechanism of excited state structural reorganization: the straight and curved arrows represent optical and relaxation transitions, respectively (F).

The photophysical properties of these 0D organic metal halide hybrids were characterized using UV-vis absorption spectroscopy, as well as steady state and time-resolved emission spectroscopy. The major photophysical properties are summarized in Table 1. Fig. 2A shows the images of the 0D organic metal halide hybrids under ambient light and UV lamp irradiation (365 nm). Highly luminescent yellow, red and orange emissions under UV irradiation were observed for (C_4_N_2_H_14_Br)_4_SnBr_6_, (C_4_N_2_H_14_I)_4_SnI_6_ and (C_9_NH_20_)_2_SbCl_5_, respectively, with the excitation and emission spectra shown in Fig. 2B. The absorption spectra of these 0D organic metal halide hybrids match well with their excitation spectra, except for the scattering in the low energy regions (Fig. S4†). The excitation maxima shift from 355 nm to 410 nm upon substitution of Br with I in the SnX_6_^4–^ octahedron, consistent with the weaker ligand field effect of I *versus* Br. The emissions of these 0D organic metal halide hybrids show extremely large Stokes shifts (>200 nm) and full widths at half maximum (FWHM) (>100 nm) which are similar to those observed in rare-earth doped phosphors with localized excited states.^13^ To verify these emissions representing the intrinsic properties of the 0D organic metal halide hybrids, we have measured the dependence of emission intensity on excitation power at room temperature. As shown in Fig. 2C, the intensity of the broadband yellow emission from (C_4_N_2_H_14_Br)_4_SnBr_6_ exhibits a linear dependence on the excitation power up to 500 W cm^–2^, suggesting that the emission does not arise from permanent defects.^14^ The emissions of these materials become narrower at 77 K (Fig. 2D), which is likely attributed to the reduced thermally populated vibrational states at low temperature. The decay curves of the broadband emissions from these 0D organic metal halide hybrids at room temperature and 77 K are shown in Fig. 2E, giving long lifetimes of ∼2.2 μs for (C_4_N_2_H_14_Br)_4_SnBr_6_, ∼1.1 μs for (C_4_N_2_H_14_I)_4_SnI_6_ and ∼4.2 μs for (C_9_NH_20_)_2_SbCl_5_. Such long lifetimes, similar to those of phosphorescence from many heavy metal complexes, suggest that the emissions are likely from the triplet states.^3^ The similar decay behaviors at room temperature and 77 K suggest little-to-no change of the characteristics of the radiative and non-radiative processes. These 0D organic metal halide hybrids possess extremely high PLQEs at room temperature: 95 ± 5% for (C_4_N_2_H_14_Br)_4_SnBr_6_, 75 ± 4% for (C_4_N_2_H_14_I)_4_SnI_6_ and 98 ± 2% for (C_9_NH_20_)_2_SbCl_5_ (Fig. S5†), and are among the most efficient light emitters developed to date. These 0D organic metal halide hybrids also showed great stability under continuous high power mercury lamp irradiation (150 mW cm^–2^) (Fig. S6†), as well as high thermal stability (Fig. S7†). Thermogravimetric analysis (TGA) shows that Sn based 0D organic metal halide hybrids do not decompose until 200 °C (Fig. S8†). This high stability in air is not surprising if we consider the unique core–shell structure, having the photoactive metal halide species well protected by the organic shells.

**Table 1:** Photophysical properties of 0D organic metal halide hybrids at room temperature and 77 K^*a*^

Material	*λ*_exc_ (nm)	*λ*_em_ (nm)	FWHM (nm)	Stokes shift (nm)	*φ* (%)	*τ*_av_ (μs)
(C_4_N_2_H_14_Br)_4_SnBr_6_	355	570 (530)	105 (63)	215	95 ± 5	2.2 (1.8)
(C_4_N_2_H_14_I)_4_SnI_6_	410	620 (626)	118 (63)	210	75 ± 4	1.1 (1.1)
(C_9_NH_20_)_2_SbCl_5_	380	590 (592)	119 (77)	210	98 ± 2	4.2 (4.7)

^*a*^ *λ*_exc_ is the wavelength at the excitation maximum; *λ*_em_ is the wavelength at the emission maximum; *φ* is the photoluminescence quantum efficiency; *τ*_av_ is the photoluminescence lifetime; the values in parentheses are for 77 K.

The broadband emissions with large Stokes shifts suggest that they are not from the direct excited states, but from other lower energy excited states. As these 0D organic metal halide hybrids are indeed perfect host–guest systems with luminescent molecular species periodically embedded in an inert matrix without intermolecular interactions or band formation, the emissions of the bulk materials are therefore from the individual metal halide molecular species, SnX_6_^4–^ and SbX_5_^2–^. Molecular excited state structural reorganization is a well-known mechanism accounting for large Stokes shifts for a number of light emitting materials,^15^–^19^ including the Sn bromide complex [NEt_4_]SnBr_3_ in solution, with characteristic metal-centered sp transitions. Therefore, the excited state processes for these 0D organic metal halide hybrids can be depicted in the configuration coordinate diagram given in Fig. 2F. Upon photon absorption, the metal halide species are excited to the high energy excited states, which undergo ultrafast excited state structural reorganization to the lower energy excited states, to generate strongly Stokes shifted broadband emissions with lifetimes of microseconds. On the other hand, similar below gap broadband emissions have been observed in corrugated-2D and 1D metal halide perovskites, as a result of exciton self-trapping.^14^,^20^–^22^ It is well-known for metal halides that the formation of localized self-trapped excited states is critically dependent on the dimensionality of the crystalline systems, and lowering the dimensionality makes exciton self-trapping easier.^23^–^25^ Therefore, 0D systems with the strongest confinement are reasonably expected to be favorable for the formation of self-trapped excited states. Indeed, the yellow emission from (C_4_N_2_H_14_Br)_4_SnBr_6_ is very similar to the self-trapped 2.2 eV emission from SnBr_2_ crystals at low temperature (12 K).^26^ Unlike corrugated 2D and 1D perovskites, with electronic band formation due to the connections between the metal halide octahedra and structural distortions, emitting from both free excitons and self-trapped excited states at room temperature,^14^,^20^–^22^ these Sn based 0D perovskites (C_4_N_2_H_14_X)_4_SnX_6_ without band formation emit from the indirect reorganized excited states only. Therefore, these 0D organic metal halide hybrids allow us to relate the classic solid-state theory of “exciton self-trapping” to the molecular photophysics term of “excited state structural reorganization”, as the metal halide building blocks could be considered as either “crystal lattice points” or “molecular species”. It should be pointed out that the true 0D perovskites (C_4_N_2_H_14_X)_4_SnX_6_ presented here are fundamentally different from previously reported analogous compounds, such as Cs_4_PbBr_6_ and Cs_2_SnI_6_, which possess little-to-no confinement of the individual metal halide octahedra and exhibit emissions from the direct excited sates.^27^–^32^

**Fig. 3:**
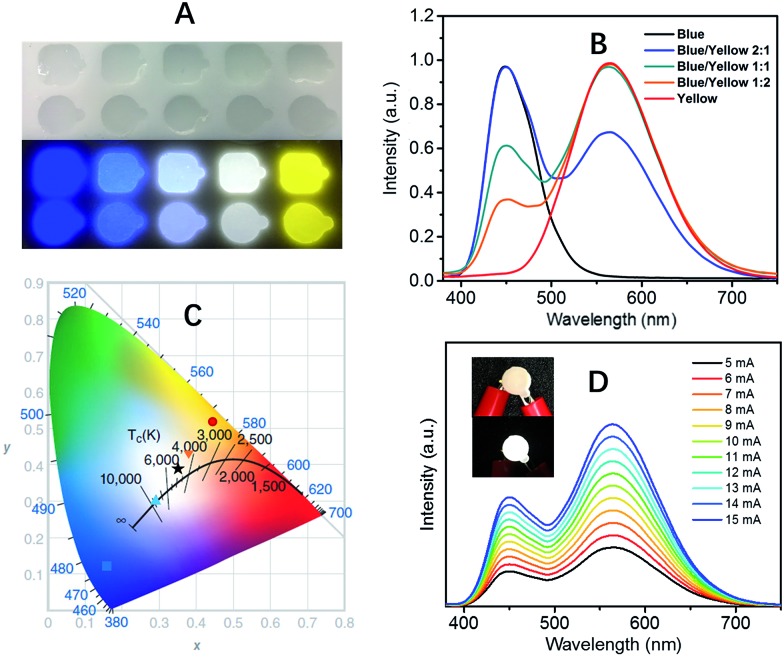
(C_4_N_2_H_14_Br)_4_SnBr_6_ as a yellow phosphor for UV pumped white LEDs. (A) Images of blue phosphors, yellow phosphors, and their blends with different weight ratios (1 : 2, 1 : 1, and 2 : 1) embedded in PDMS under ambient light (top) and irradiation from a hand held UV lamp (365 nm) (bottom). (B) Emission spectra of UV pumped LEDs with different blending ratios of the blue and yellow phosphors. (C) CIE coordinates and CCTs for the UV pumped LEDs plotted on the CIE1931 chromaticity chart: blue (■), “cold” white (▲), white (★), “warm” white () and yellow (). (D) Emission spectra of a white LED at different driving currents, the insets show the device when off and on.

The extremely high PLQEs in the solid state make these earth-abundant lead-free materials highly promising light emitters for a variety of applications. Unlike many light emitters, such as organic emitters and colloidal quantum dots which require doping to prevent aggregation induced self-quenching in the solid state, these 0D organic metal halide hybrids are perfect host–guest systems themselves. The strongly Stokes shifted broadband emissions without self-absorption are of particular interest for applications in down conversion white LEDs and luminescent solar concentrators. To demonstrate the application of these materials as phosphors, we fabricated down conversion LEDs, in which a commercial UV LED (340 nm) was used to optically pump polydimethylsiloxane (PDMS) films blended with ball-milled yellow emitting (C_4_N_2_H_14_Br)_4_SnBr_6_ and commercial blue emitting europium-doped barium magnesium aluminates (BaMgAl_10_O_17_:Eu^2+^).^33^ The UV LED (340 nm) was chosen considering the excitations of both the yellow and blue phosphors in the UV region (Fig. S9†). Fig. 3A shows images of PDMS films doped with blue and yellow phosphors at different weight ratios under ambient light and UV lamp irradiation. The emission spectra of the UV pumped LEDs are shown in Fig. 3B. The CIE color coordinates and Correlated Color Temperatures (CCTs) are shown in Fig. 3C. A nice range of “cold” to “warm” white light has been achieved by controlling the blending ratio between the two phosphors. With a blue/yellow weight ratio of 1 : 1, a decent white emission, with CIE coordinates of (0.35, 0.39), a CCT of 4946 K and a color-rendering index (CRI) of 70, was obtained. Excellent color stability was observed in this white LED at different operating currents, as shown in Fig. 3D. This could be attributed to the little-to-no energy transfer from the blue phosphors to the yellow phosphors, as there is minimal overlap between the excitation of the yellow phosphors and the emission of the blue phosphors (Fig. S9†). The white LED also showed great stability in air with almost no change of light brightness and color during the preliminary testing, *i.e.* the device was continuously on at ∼400 cd m^–2^ for more than six hours under the same operating power (Fig. S10†).

## Conclusions

By using appropriate organic and inorganic building blocks, we have been able to assemble a series of 0D organic–inorganic hybrid materials containing photoactive metal halide species, which represent perfect host–guest systems with the metal halide species periodically embedded in wide band gap organic networks through ionic bonds. Without electronic band formation between the individual metal halide species or quantum size effects, these ionically bonded 0D hybrid materials enable access to the intrinsic properties of individual photoactive molecular species in bulk materials, opening up routes towards the formation of a new generation of high performance light emitting materials for optoelectronic devices.

## Experimental

### Materials

Tin(ii) bromide, tin(ii) iodide, antimony trichloride, *N*,*N*′-dimethylethylenediamine (99%), 1-butyl-1-methylpyrrolidinium chloride, γ-butyrolactone (GBL, ≥99%), hydrobromic acid (48 wt% in H_2_O) and hydriodic acid (55%) were purchased from Sigma-Aldrich. Dichloromethane (DCM, 99.9%), dimethylformamide (DMF, 99.8%), toluene (anhydrous, 99.8%) and ethyl ether (stabilized with ∼1 ppm BHT) were purchased from VWR. Acetone (HPLC grade) was purchased from EMD Millipore. All reagents and solvents were used without further purification unless otherwise stated.

### *N*,*N*′-Dimethylethylene-1,2-diammonium halide salts

*N*,*N*′-dimethylethylene-1,2-diammonium bromide salts were prepared by adding hydrobromic acid solution (2.2 equiv.) into *N*,*N*′-dimethylethylenediamine (1 equiv.) in ethanol at 0 °C. The organic salts were obtained after the removal of the solvents and starting reagents under vacuum, followed by washing with ethyl ether. The salts were dried and kept in a desiccator for future use. *N*,*N*′-Dimethylethylene-1,2-diammonium iodide salts were prepared following a similar method.

### Solution growth of single crystalline 0D Sn halide bulk materials

Tin(ii) bromide and *N*,*N*′-dimethylethylene-1,2-diammonium bromide were mixed in a 1 : 4 molar ratio and dissolved in DMF to form a clear precursor solution. Bulk crystals were prepared by diffusing DCM into DMF solution at room temperature overnight. The large colorless crystals were washed with acetone and dried under reduced pressure. The yield was calculated to be ∼70%. C_16_H_56_N_8_SnBr_10_: anal. calc. C, 15.03; H, 4.42; N, 8.77. Found: C, 15.31; H, 4.24; N, 8.74. Tin(ii) iodide and *N*,*N*′-dimethylethylene-1,2-diammonium iodide were mixed in a 1 : 4 molar ratio and dissolved in GBL to form a clear precursor solution. Bulk crystals were prepared by diffusing DCM into GBL solution at room temperature overnight. The large reddish crystals were washed with acetone and dried under reduced pressure. The yield was calculated to be ∼70%. C_16_H_56_N_8_SnI_10_: anal. calc. C, 10.99; H, 3.23; N, 6.41. Found: C, 11.16; H, 3.23; N, 6.24.

### Solution growth of single crystalline 0D Sb halide bulk materials

Antimony(ii) chloride and 1-butyl-1-methylpyrrolidinium chloride were mixed in a 1 : 2 molar ratio and dissolved in DMF to form a clear precursor solution. Bulk crystals were prepared by diffusing acetone into DMF solution at room temperature overnight. The colorless crystals were washed with acetone and dried under reduced pressure. The yield was calculated to be ∼70%. C_18_H_40_N_2_SbCl_5_: anal. calc. C, 37.05; H, 6.91; N, 4.80. Found: C, 37.32; H, 6.84; N, 4.83.

### Single crystal X-ray diffraction (SCXRD)

Single crystal X-ray diffraction data of (C_4_N_2_H_14_Br)_4_SnBr_6_ were collected using an Oxford-Diffraction Xcalibur-2 CCD diffractometer with graphite-monochromated Mo Kα radiation. The crystal was mounted in a cryoloop under Paratone-N oil and cooled to 120 K with an Oxford-Diffraction Cryojet. A complete sphere of data was collected using ω scans with 1° frame widths to a resolution of 0.6 Å, equivalent to 2*θ* ≈ 72.5°. Reflections were recorded, indexed and corrected for absorption using the Oxford-Diffraction CrysAlisPro software, and subsequent structure determination and refinement was carried out using CRYSTALS, employing superflip to solve the crystal structure. The data did not allow for an unconstrained refinement: all hydrogens were restrained to the connecting nitrogen or carbon. The refinement was performed against *F*^2^, with anisotropic thermal displacement parameters for all non-hydrogen atoms and with isotropic thermal displacement parameters for the hydrogens in the structure. The crystal structure of (C_9_NH_20_)_2_SbCl_5_ was resolved using the same method. (C_4_N_2_H_14_I)_4_SnI_6_ was mounted on a nylon loop with the use of heavy oil. The sample was held at 100 K for data collection. The data were recorded on a Bruker SMART APEX II diffractometer using a detector distance of 6 cm. The number of frames taken was 2400 using 0.3 degree omega scans with either 20 or 30 seconds of frame collection time. Integration was performed using the program SAINT which is part of the Bruker suite of programs. Absorption corrections were made using SADABS. XPREP was used to obtain an indication of the space group and the structure was typically solved by direct methods and refined by SHELXTL. The non-hydrogen atoms were refined anisotropically. VESTA was used as the crystal structure visualization software for the images presented in the manuscript.

### Powder X-ray diffraction (PXRD)

The PXRD analysis was performed on a Panalytical X’PERT Pro Powder X-ray Diffractometer using copper X-ray tube (standard) radiation at a voltage of 40 kV and 40 mA, and an X’Celerator RTMS detector. The diffraction pattern was scanned over the angular range of 5–50 degrees (2*θ*) with a step size of 0.02, at room temperature. Simulated powder patterns were calculated by Mercury software using the crystallographic information files (CIFs) from single-crystal X-ray experiments.

### Sn nuclear magnetic resonance (NMR) spectroscopy

The ^119^Sn MAS NMR spectra were recorded on a Bruker Advance III HD spectrometer equipped with a 4 mm MAS probe, operating at 186.5 MHz with the samples spinning at 12 kHz, high power proton decoupling, a 30 s recycle delay, and typically 2048 scans. SnO_2_ was used as a secondary reference at –604.3 ppm.

### X-ray photoelectron spectroscopy (XPS)

XPS measurements were carried out using a ULVACPHI, Inc., PHI 5000 VersaProbe II. The survey XPS spectra were recorded with a monochromatic Al Kα source using a 93.9 pass energy and 0.8 eV per step. High-resolution spectra were recorded using a 11.75 pass energy and 0.1 eV per step. The high-resolution spectra binding energies were assigned using a C 1s binding energy of 286.2 eV for the C–N= bonds in (C_4_N_2_H_14_Br)_4_SnBr_6_. A binding energy of 487.0 eV for Sn 3d^5^ was then found to correspond to that of Sn(ii) in SnBr_2_.

### Absorption spectrum measurement

Absorption spectra of 0D organic metal halide hybrids were measured at room temperature through synchronous scans in an integrating sphere incorporated into the spectrofluorometer (FLS980, Edinburgh Instruments) while maintaining a 1 nm interval between the excitation and emission monochromators.

### Excitation spectrum measurement

Excitation spectra of the 0D organic metal halide hybrids were measured at room temperature on a FLS980 spectrofluorometer (Edinburgh Instruments) monitored at the maximum of the emission spectra.

### Steady-state photoluminescence studies

Steady-state photoluminescence spectra of the 0D organic metal halide hybrids were obtained at room temperature and 77 K (liquid nitrogen was used to cool the samples) on a FLS980 spectrofluorometer.

### Temperature dependent photoluminescence

The temperature dependent photoluminescence spectra were measured on a Varian Cary Eclipse Fluorescence Spectrometer with a Water 4 Position Multicell Holder Accessory attached to a Julabo F12-EC Refrigerated/Heating Circulator filled with ethylene glycol-water mixture (3 : 2). It is interesting to see that the PL intensity of 0D (C_4_N_2_H_14_Br)_4_SnBr_6_ bulk crystals even increases a bit upon an increase in temperature (recovering upon a decrease in temperature), which is likely due to the change of refractive index of the bulk crystal samples (absorption increasing upon an increase in temperature).

### Photoluminescence quantum efficiencies (PLQEs)

For photoluminescence quantum efficiency measurements, the samples were excited using a light output from a housed 450 W Xe lamp passed through a single grating (1800 L mm^–1^, 250 nm blaze) Czerny–Turner monochromator and finally a 5 nm bandwidth slit. Emission from the sample was passed through a single grating (1800 L mm^–1^, 500 nm blaze) Czerny–Turner monochromator (5 nm bandwidth) and detected by a Peltier-cooled Hamamatsu R928 photomultiplier tube. The absolute quantum efficiencies were acquired using an integrating sphere incorporated into an FLS980 spectrofluorometer. The PLQE was calculated by the equation: *η*_QE_ = *I*_S_/(*E*_R_ – *E*_S_), in which *I*_S_ represents the luminescence emission spectrum of the sample, *E*_R_ is the spectrum of the excitation light from the empty integrated sphere (without the sample), and *E*_S_ is the excitation spectrum for exciting the sample. The control samples, rhodamine 101 and the blue phosphor BaMgAl_10_O_17_:Eu^2+^, were measured using this method to give PLQEs of ∼98% and ∼93%, which are close to the literature reported values. The PLQEs were doubly confirmed by a Hamamatsu C9920 system equipped with a xenon lamp, calibrated integrating sphere and model C10027 photonic multi-channel analyzer (PMA). The measurements taking account of indirect PL provided the same results within the error bars.

### Time-resolved photoluminescence

Time-resolved emission data were collected at room temperature and 77 K (liquid nitrogen was used to cool the samples) using time-correlated single photon counting on a Horiba JY Fluoromax-4 Fluorometer. The samples were excited with 295 nm pulsed diode lasers. Emission counts were monitored at 530 nm. The average lifetime was obtained by multiexponential fitting.

### Photoluminescence intensity dependence on the excitation power density

The PL intensity *versus* power studies were carried out on an Edinburgh Instruments PL980-KS transient absorption spectrometer using a Continuum Nd:YAG laser (Surelite EX) pumping a Continuum Optical Parametric Oscillator (Horizon II OPO) to provide 360 nm 5 ns pulses at 1 Hz. The pump beam profile was carefully defined using collimated laser pulses passed through an iris set to a 5 mm diameter. The pulse intensity was monitored by a power meter (Ophir PE10BF-C) detecting the reflection from a beam splitter. The power meter and neutral density filters were calibrated using an identical power meter placed at the sample position. Neutral density filters and an external power attenuator were used to reduce the power density of the pump to the desired power range. Detection consisted of an Andor intensified CCD (1024 × 256 element) camera collecting a spectrum from 287 nm to 868 nm and gated to optimize PL collection (typically a 30 to 50 ns gate depending on the PL lifetime, starting immediately following the 5 ns laser pulse). 100 collections were averaged at each power level with every laser pulse monitored to determine the average intensity. The PL intensity was determined at the maximum of the PL emission curve.

### Material photostability study

To test the photostability, a 100 W 20 V mercury short arc lamp was used as a continuous irradiation light source. The intensity of the irradiation was calibrated to 150 mW cm^–2^. The emission was measured periodically on a HORIBA iHR320 spectrofluorimeter, equipped with a HORIBA Synapse CCD detection system.

### Thermogravimetric analysis (TGA)

TGA was carried out using a TA instruments Q50 TGA system. The samples were heated from room temperature (around 22 °C) to 800 °C at a rate of 5 °C min^–1^, under a nitrogen flux of 100 mL min^–1^.

### UV pumped LEDs

The blue (BaMgAl_10_O_17_:Eu^2+^) and yellow ((C_4_N_2_H_14_)_4_SnBr_10_) phosphors were blended with a Sylgard 184 polydimethylsiloxane (PDMS) encapsulant, and put in a polytetrafluoroethylene (PTFE) mold to control the shape and thickness. The whole mold was heated at 100 °C for 40 min in an oven to cure PDMS. The phosphor doped PDMS films were then attached to a UVTOP® UV LED with a window, 340 nm, 0.33 mW (THORLABS) to form UV pumped LEDs. The LEDs were driven by a Keithley 2400 sourcemeter and emission spectra were recorded on an Ocean Optics USB4000 Miniature Fiber Optic Spectrometer. For the device stability test, a white light LED was continuously powered by a Keithley 2400 at a stable current power to give a brightness of ∼400 cd m^–2^. Emission spectra were recorded at periodic intervals using an Ocean Optics USB4000 Miniature Fiber Optic Spectrometer.

## Conflicts of interest

There are no conflicts to declare.

## Supplementary Material

Supplementary information

Crystal structure data

## References

[cit1] Tang C. W., Vanslyke S. A. (1987). Appl. Phys. Lett..

[cit2] Burroughes J. H., Bradley D. D. C., Brown A. R., Marks R. N., Mackay K., Friend R. H., Burn P. L., Holmes A. B. (1990). Nature.

[cit3] Baldo M. A., O’Brien D. F., You Y., Shoustikov A., Sibley S., Thompson M. E., Forrest S. R. (1998). Nature.

[cit4] Lin C. C., Liu R. S. (2011). J. Phys. Chem. Lett..

[cit5] Colvin V. L., Schlamp M. C., Alivisatos A. P. (1994). Nature.

[cit6] Tan Z. K., Moghaddam R. S., Lai M. L., Docampo P., Higler R., Deschler F., Price M., Sadhanala A., Pazos L. M., Credgington D., Hanusch F., Bein T., Snaith H. J., Friend R. H. (2014). Nat. Nanotechnol..

[cit7] Tang C. W., Vanslyke S. A., Chen C. H. (1989). J. Appl. Phys..

[cit8] Callaway J., Quantum theory of the solid state, Academic Press, 2013.

[cit9] Norris D., Bawendi M. (1996). Phys. Rev. B: Condens. Matter Mater. Phys..

[cit10] Siegrist T., Kloc C., Laudise R. A., Katz H. E., Haddon R. C. (1998). Adv. Mater..

[cit11] Mitzi D. B. (2001). J. Chem. Soc., Dalton Trans..

[cit12] Saparov B., Mitzi D. B. (2016). Chem. Rev..

[cit13] Tucureanu V., Matei A., Avram A. M. (2015). Opto-Electron. Rev..

[cit14] Dohner E. R., Jaffe A., Bradshaw L. R., Karunadasa H. I. (2014). J. Am. Chem. Soc..

[cit15] Miller M. T., Gantzel P. K., Karpishin T. B. (1998). Inorg. Chem..

[cit16] Shaw G. B., Grant C. D., Shirota H., Castner E. W., Meyer G. J., Chen L. X. (2007). J. Am. Chem. Soc..

[cit17] Mel’nikov M. Y., Weinstein J. A. (2008). High Energy Chem..

[cit18] Zhou C., Tian Y., Yuan Z., Han M., Wang J., Zhu L., Tameh M. S., Huang C., Ma B. (2015). Angew. Chem., Int. Ed..

[cit19] Oldenburg K., Vogler A. (1993). Z. Naturforsch., B: J. Chem. Sci..

[cit20] Hu T., Smith M. D., Dohner E. R., Sher M. J., Wu X. X., Trinh M. T., Fisher A., Corbett J., Zhu X. Y., Karunadasa H. I., Lindenberg A. M. (2016). J. Phys. Chem. Lett..

[cit21] Cortecchia D., Yin J., Bruno A., Lo S. Z., Gurzadyan G. G., Mhaisalkar S., Bredas J. L., Soci C. (2017). J. Mater. Chem. C.

[cit22] Yuan Z., Zhou C., Shu Y., Tian Y., Messier J., Wang J., Burgt L., Kountouriotis K., Xin Y., Holt E., Schanze K. S., Clark R., Siegrist T., Ma B. (2017). Nat. Commun..

[cit23] Williams R. T., Song K. S. (1990). J. Phys. Chem. Solids.

[cit24] Georgiev M., Mihailov L., Singh J. (1995). Pure Appl. Chem..

[cit25] Wu X. X., Trinh M. T., Niesner D., Zhu H. M., Norman Z., Owen J. S., Yaffe O., Kudisch B. J., Zhu X. Y. (2015). J. Am. Chem. Soc..

[cit26] Yamasaki Y., Ohno N. (2001). Int. J. Mod. Phys. B.

[cit27] Takeoka Y., Asai K., Rikukawa M., Sanui K. (2005). Chem. Lett..

[cit28] Nikl M., Mihokova E., Nitsch K., Somma F., Giampaolo C., Pazzi G. P., Fabeni P., Zazubovich S. (1999). Chem. Phys. Lett..

[cit29] Wang A. F., Yan X. G., Zhang M., Sun S. B., Yang M., Shen W., Pan X. Q., Wang P., Deng Z. T. (2016). Chem. Mater..

[cit30] Saparov B., Sun J. P., Meng W. W., Xiao Z. W., Duan H. S., Gunawan O., Shin D., Hill I. G., Yan Y. F., Mitzi D. B. (2016). Chem. Mater..

[cit31] Akkerman Q. A., Park S., Radicchi E., Nunzi F., Mosconi E., De Angelis F., Brescia R., Rastogi P., Prato M., Manna L. (2017). Nano Lett..

[cit32] Ling Y. C., Tan L., Wang X., Zhou Y., Xin Y., Ma B. W., Hanson K., Gao H. W. (2017). J. Phys. Chem. Lett..

[cit33] Lu C. H., Hsu W. T., Huang C. H., Godbole S. V., Cheng B. M. (2005). Mater. Chem. Phys..

