# Supplementary material for: Luminescent zero-dimensional organic metal halide hybrids with near-unity quantum efficiency
Source: Chem Sci. 2017 Nov 21;9(3):586–93. doi: 10.1039/c7sc04539e (PMC5870054; doi:10.1039/c7sc04539e)
Supplement: Supplementary file 1 [file SC-009-C7SC04539E-s001.pdf]

Electronic Supplementary Material (ESI) for Chemical Science.
This journal is © The Royal Society of Chemistry 2017

Supporting Information

Luminescent Zero-Dimensional Organic Metal Halide Hybrids with Near-Unity Quantum Efficiency

Chenkun Zhou[a], Haoran Lin[a], Yu Tian[b], Zhao Yuan[a], Ronald Clark[b], Banghao Chen[b], Lambertus J. van de Burgt[b], Jamie C. Wang[b], Yan Zhou[b], Kenneth Hanson[bc], Quinton J. Meisner[b], Jennifer Neu[d], Tiglet Besara[d], Theo Siegrist[abd], Eric Lambers[e], Peter Djurovich[f], Biwu Ma[abc*]

a. *Department of Chemical and Biological Engineering, FAMU-FSU College of Engineering, Tallahassee, FL 32310, USA.*

b. *Materials Science and Engineering Program, Florida State University, Tallahassee, FL 32306, USA.*

c. *Department of Chemistry and Biochemistry, Florida State University, Tallahassee, FL 32306, USA.*

d. *National High Magnetic Field Laboratory, Florida State University, Tallahassee, FL 32310, USA.*

e. *Research Service Centers, University of Florida, Gainesville, Florida 32661, USA.*

f. *Department of Chemistry, University of Southern California, Los Angeles, California 90089, USA.*

Table S1 | Single crystal x-ray diffraction data and collection parameters. The collection was performed at a temperature of ~ 120 K.

**Compound**	**$(C_4N_2H_{14}Br)_4SnBr_6$**	**$(C_4N_2H_{14}I)_4SnI_6$**	**$(C_9NH_{20})_2SbCl_5$**
Formula	$[(CH_3NH_2)_2C_2H_4]_4SnBr_{10}$	$[(CH_3NH_2)_2C_2H_4]_4SnI_{10}$	$C_{18}H_{40}Cl_5N_2Sb$
Molecular weight	1278.40 g/mol	1748.37 g/mol	583.54 g/mol
Space group	*P* -1 (# 2)	*P* -1	$P\ 2_1/n$
a	10.2070(4) Å	10.7464(7) Å	8.7562(2) Å
b	10.6944(4) Å	10.8924(7) Å	27.2439(5) Å
c	18.5996(6) Å	11.1796(7) Å	10.64230(2) Å
α	94.043(3)°	64.2658(7) °	90.0000 °
β	102.847(3)°	80.1825(7) °	97.354(2) °
γ	97.904(3)°	72.8331(7) °	90.0000 °
V	1949.89(12) Å^3	1124.94(12) Å^3	2518.02(8) Å^3
Z	2	1	4
$\rho_{calc.}$	2.177 g/cm^3	2.581 g/cm^3	1.539 g/cm^3
μ	10.922 mm^{-1}	7.448 mm^{-1}	1.633 mm^{-1}
Data collection range	2.815° < θ < 34.220°	1.986° < θ < 29.408°	2.782° < θ < 31.981°
Reflections collected	57392	14019	32635
Independent reflections	11532	5710	8177
Parameters refined	540	165	395
Restraints	240	0	174
R_1, wR_2	0.0651^a, 0.0511^b	0.0178, 0.0349	0.0418, 0.0542
Goodness-of-fit on F^2	0.9933	1.078	0.9984

$^{a)}$ $R_1 = \Sigma \| |F_o| - |F_c| \| / \Sigma |F_o|$. $^{b)}$ $wR_2 = [\Sigma w(F_o^2 - F_c^2)^2 / \Sigma w(F_o^2)^2]^{1/2}$.

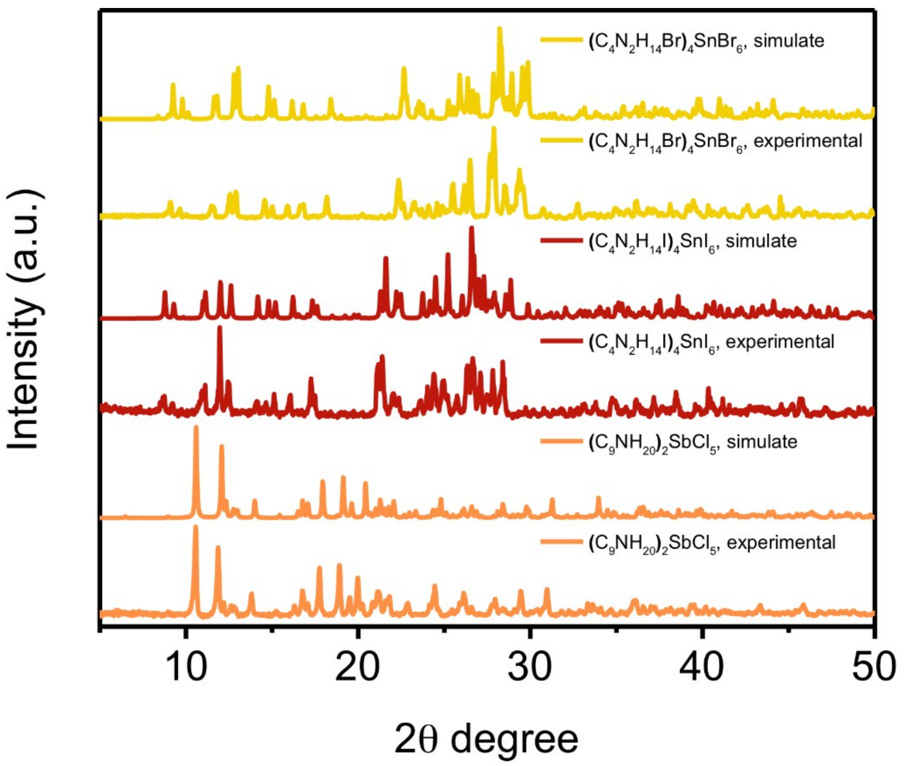

Figure S1. PXRD of powder 0D organic metal halide hybrids as well as their simulated results.

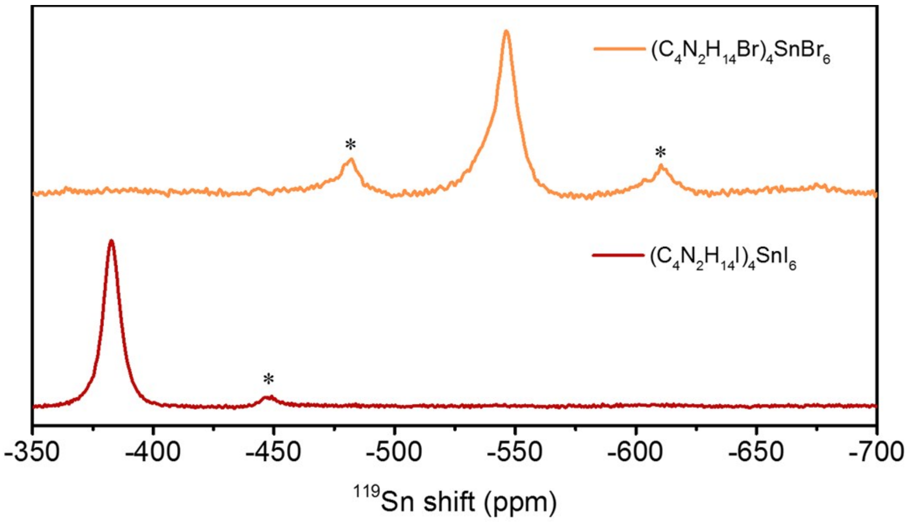

Figure S2. ^{119}Sn MAS NMR spectra of 0D organic tin halide hybrids recorded at room temperature spinning at 12 kHz. Spinning sidebands are indicated with asterisks.

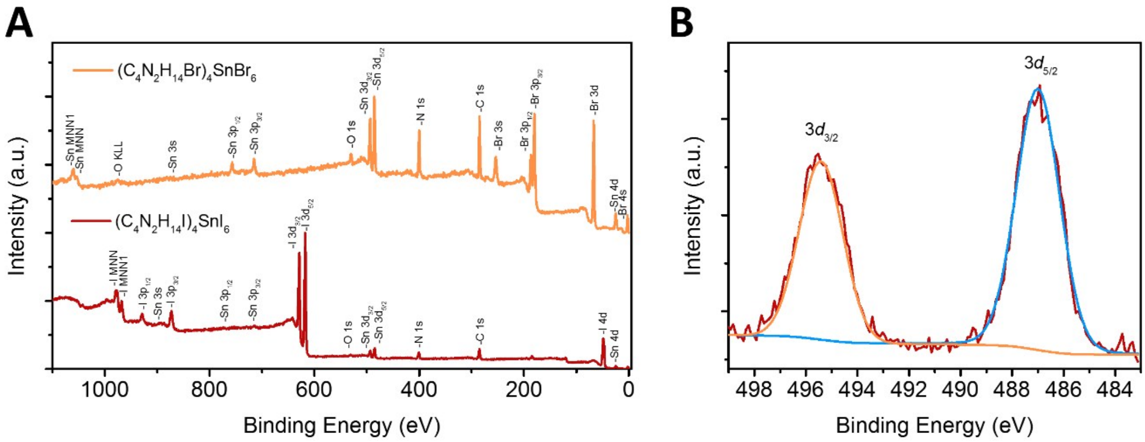

Figure S3. X-ray photoelectron spectroscopy (XPS) of 0D organic tin halide hybrids: (a) survey spectra of two 0D organic tine halide hybrids, and (b) high resolution Sn spectra in $(C_4N_2H_{14}Br)_4SnBr_6$.

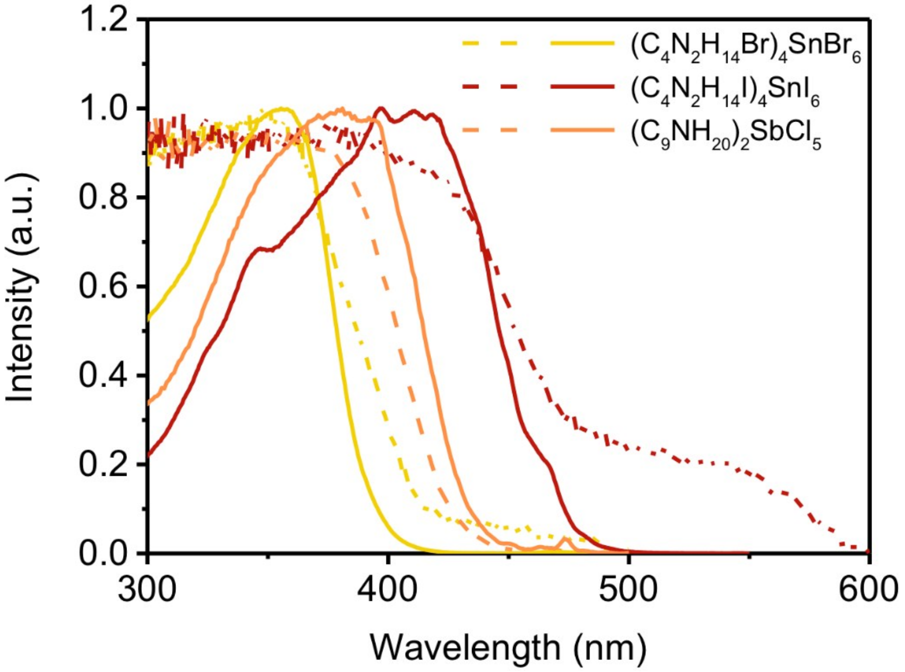

Figure S4. Absorption (dash line) and excitation (solid line) spectra of 0D organic metal halide hybrids recorded at room temperature.

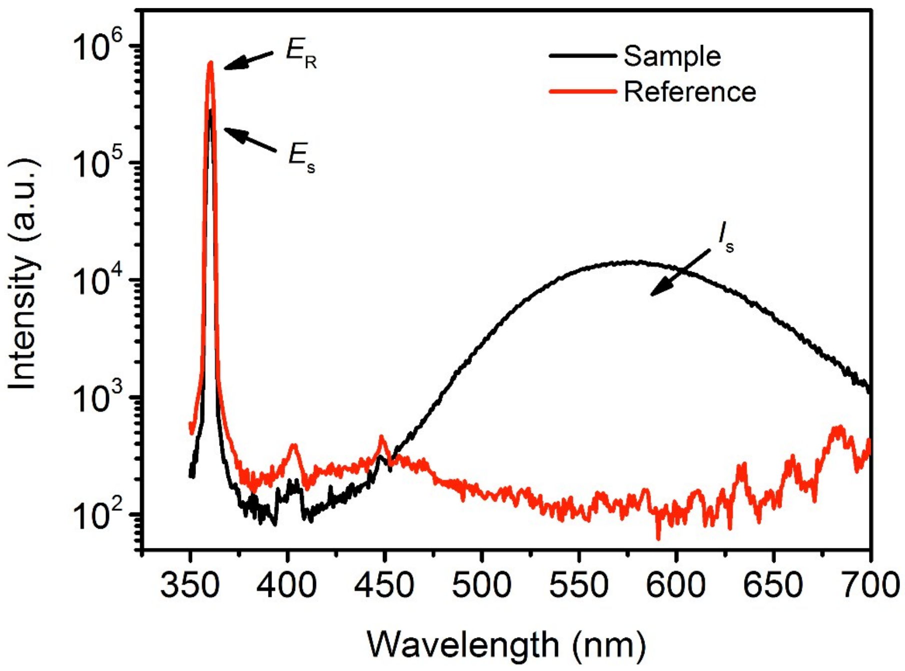

Figure S5. Excitation line of reference and emission spectrum of $(C_4N_2H_{14}Br)_4SnBr_6$ collected by an integrating sphere. The PLQE was calculated by the equation: $\eta_{QE} = I_S/(E_R - E_S)$.

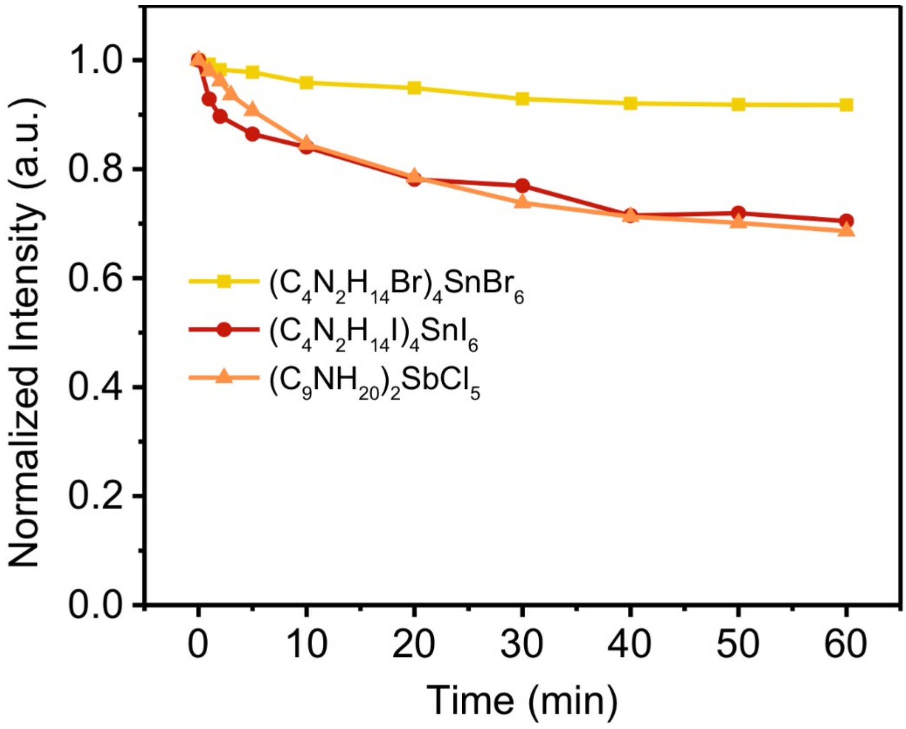

Figure S6. Photostability of 0D organic metal halide hybrids under continuous illumination using a high-power mercury lamp (150 mW/cm^2).

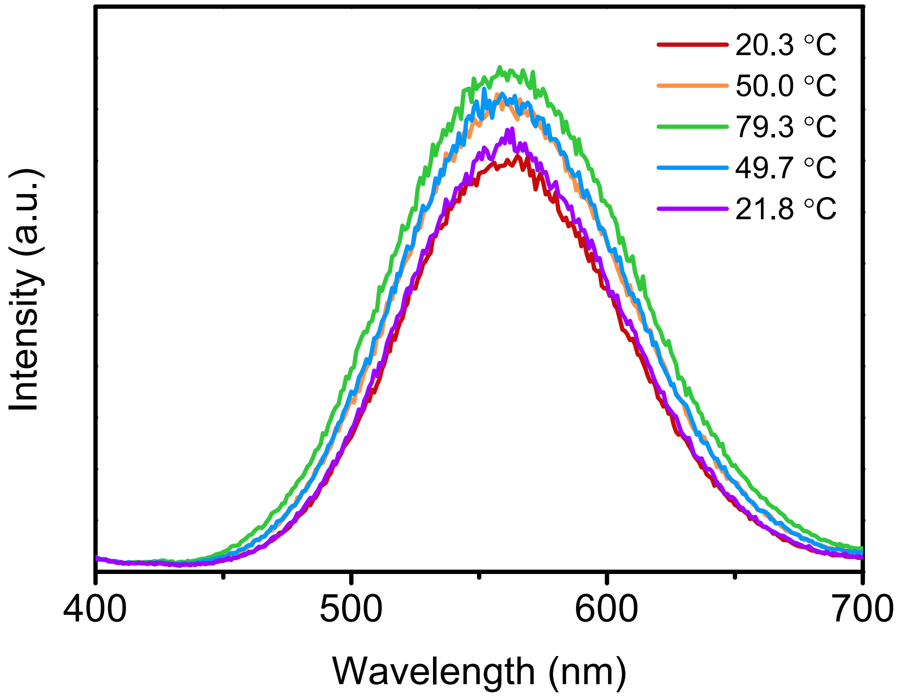

Figure S7. Temperature dependent photoluminescence of $(C_4N_2H_{14}Br)_4SnBr_6$.

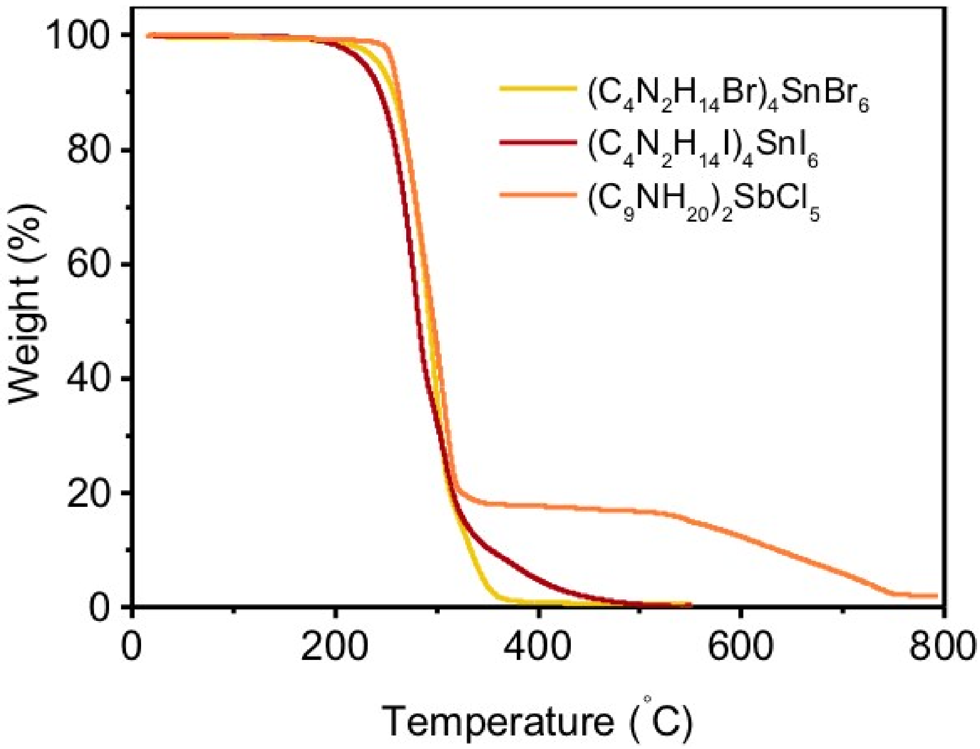

Figure S8. TGA of Sn based 0D organic metal halide hybrids.

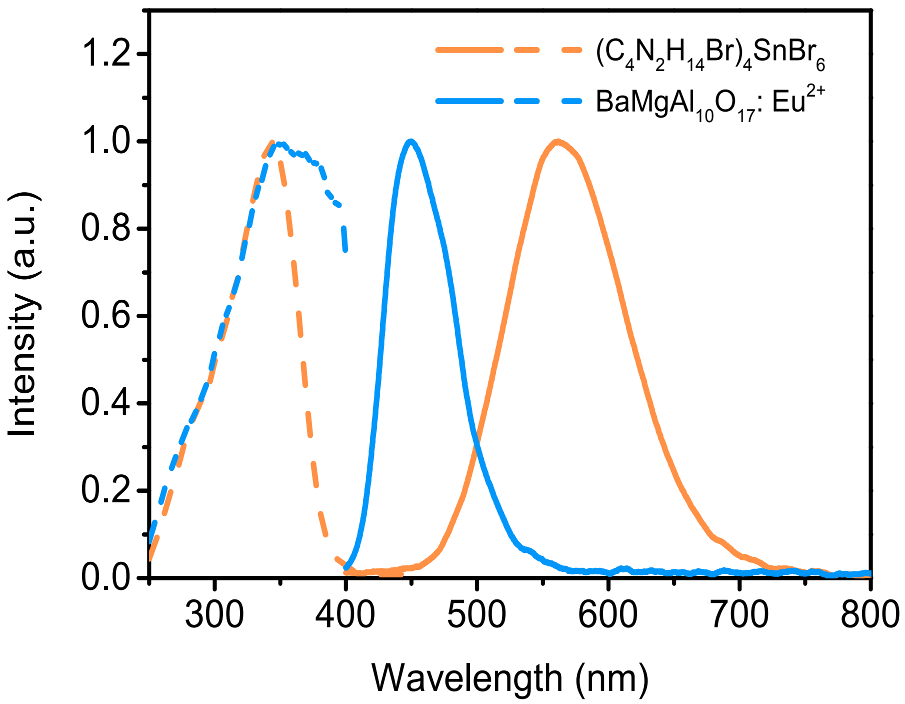

Figure S9. The normalized excitation (dash lines) and emission (solid lines) spectra of $BaMgAl_{10}O_{17}$:Eu^{2+} and $(C_4N_2H_{14}Br)_4SnBr_6$ phosphors.

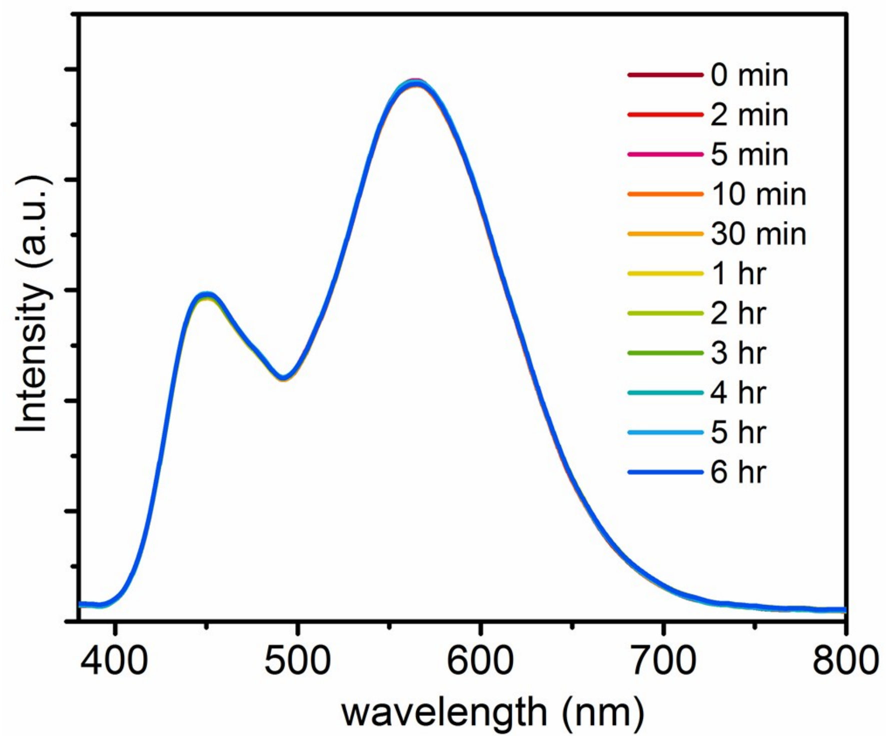

Figure S10. Emission spectra of a white LED continuously operated in air for more than six hours with a brightness of ~ 400 cd/m^2.
